# Contribution of circulating monocytes in maintaining homeostasis of resident macrophages in postnatal and young adult mouse cochlea

**DOI:** 10.1038/s41598-023-50634-y

**Published:** 2024-01-02

**Authors:** Toru Miwa, Gowshika Rengasamy, Zhaoyuan Liu, Florent Ginhoux, Takayuki Okano

**Affiliations:** 1 Department of Otolaryngology, Head and Neck Surgery, Graduate School of Medicine, Kyoto University, Sakyo-ku, Kyoto Japan; 2 Department of Otolaryngology-Head and Neck Surgery, Osaka Metropolitan University, Abeno-ku, Osaka Japan; 3 Singapore Immunology Network, Agency for Science, Technology and Research, Singapore, Singapore; 4 Shanghai Institute of Immunology, Department of Immunology and Microbiology, Shanghai Jiao Tong University School of Medicine, Shanghai, China; 5 SingHealth Duke-NUS Academic Medical Centre, Translational Immunology Institute, Singapore, Singapore; 6 Institut Gustave Roussy, INSERM U1015, Villejuif, France; 7 Department of Otolaryngology-Head and Neck Surgery, Fujita Health University, Toyoake, Aichi Japan

**Keywords:** Immunology, Neuroimmunology, Microglia

## Abstract

The percentage of macrophage subpopulations based on their origins in the adult cochlea remains unclear. This study aimed to elucidate the origins of cochlear macrophages during the onset phase and development of auditory function. We used three types of mice: wildtype ICR mice, colony-stimulating factor 1 receptor (*Csf1r*)-deficient mice, and *Ms4a3Cre-Rosa tdTomato* (*Ms4a3*^*tdT*^) transgenic mice. Macrophages were labeled with ionized calcium-binding adapter molecule 1 (Iba1), which is specific to more mature macrophages, and CD11b, which is specific to monocyte lineage. We investigated the spatial and temporal distribution patterns of resident macrophages in the cochlea during the postnatal and early adult stages. During the adult stages, the rate of monocytes recruited from the systemic circulation increased; moreover, Iba1^+^/CD11b^−^ cochlear macrophages gradually decreased with age. Fate mapping of monocytes using *Ms4a3*^*tdT*^ transgenic mice revealed an increased proportion of bone marrow-derived cochlear macrophages in the adult stage. Contrastingly, the proportion of yolk sac- and fetal liver-derived tissue-resident macrophages decreased steadily with age. This heterogeneity could be attributed to differences in environmental niches within the tissue or at the sub-tissue levels. Future studies should investigate the role of cochlear macrophages in homeostasis, inflammation, and other diseases, including infection, autoimmune, and metabolic diseases.

## Introduction

Macrophages are present in virtually all vertebrate tissues; they emerge before any other immune cell type mid-gestation and are present in almost all organs and tissues throughout life^1,2^. In addition to regulation of tissue development and regeneration, macrophages contribute to local homeostasis by responding to internal and external stimuli as well as phagocytosing microbes and their products. Further, they are involved in the clearance of purposeless and senescent cells and act as sentinels with trophic, regulatory, and repair functions. Heterogeneous macrophage phenotypes have been observed in different tissue environments, and their organ-specific roles in developmental processes and normal physiology have been highlighted^3–5^. Additionally, macrophages exhibit diverse tissue-specific functions by integrating cues from their external surroundings and microenvironment.

**Figure 1:**
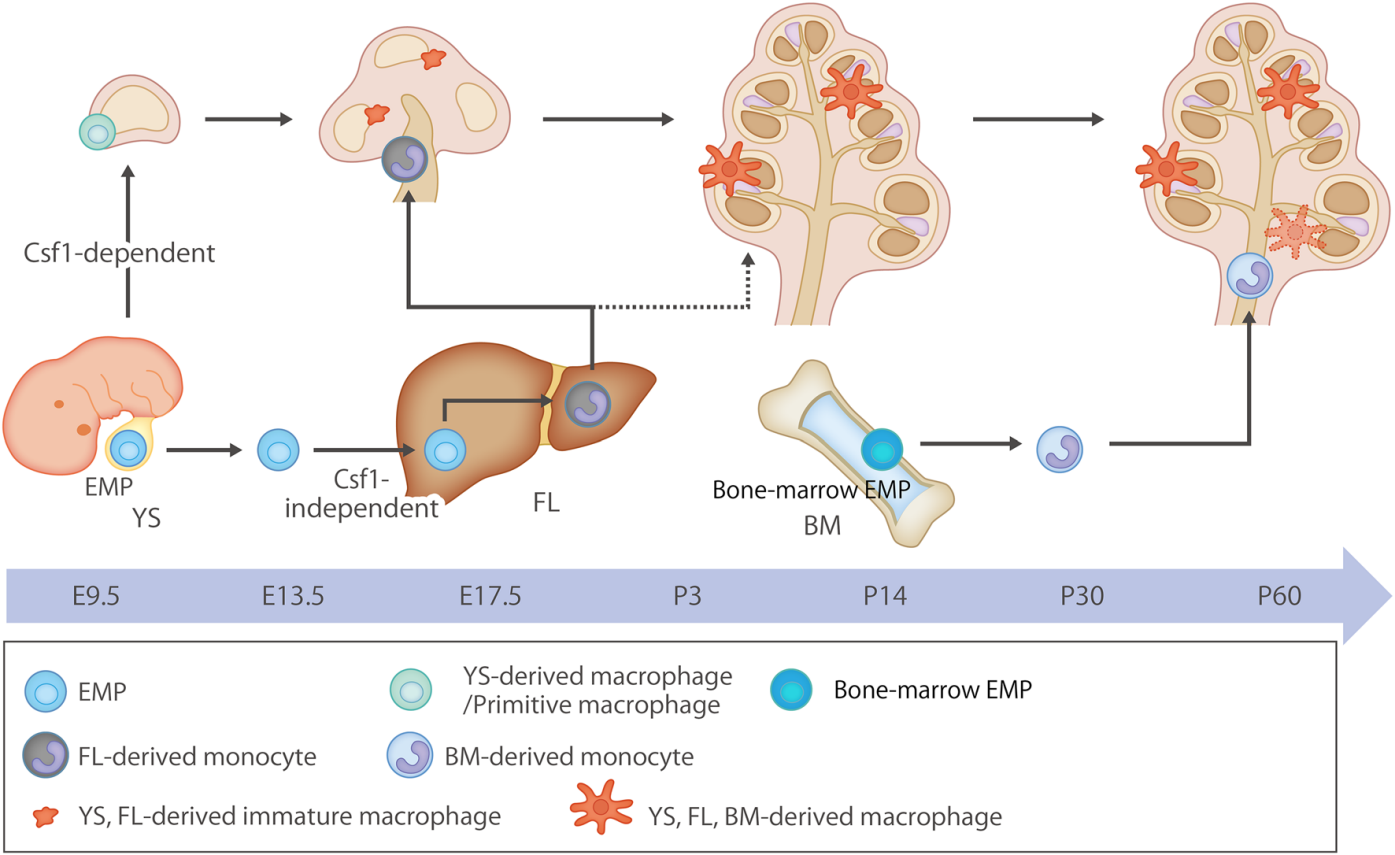
Origin and renewal of tissue-resident macrophages, schematic representation of the origins and distribution of resident macrophages in embryonic and adult cochleae, and distribution of tissue macrophages in the cochlea. Two subtypes of resident macrophages (Mϕ) are present within the embryonic cochlea: Csf1r-dependent Mϕ, which originate from the YS, and Csf1r-independent Mϕ, which migrate from the FL via systemic circulation. A large proportion of the cochlear-resident Mϕ population was derived from the YS since Iba1-positive Mϕs reside in the mesenchyme surrounding the otocyst as early as E10.5. Csf1r-independent Mϕ expressing CD11b migrate as early as E14.5 and reside only in specific components of the cochlea. In the adult cochlea, the density of Mϕs expressing CD11b increases, suggesting that FL and BM contribute to the repopulation of cochlear-resident Mϕ. YS, yolk sac; FL, fetal liver; BM, bone marrow; Mϕ, macrophage; Csf1r, colony-stimulating factor 1 receptor; EMP, erythro-myeloid progenitor.

Previously, it was unclear whether resident macrophages are constantly and predominantly repopulated by blood-circulating monocytes derived from bone marrow (BM) progenitors. However, recent studies have demonstrated that specific macrophage populations are independent of circulating monocytes and even adult BM hematopoiesis^6–9^. Instead, these tissue-resident macrophages are derived from the sequential migration and settlement of various precursors into tissues during embryonic development. Primitive macrophages generated from early erythro-myeloid progenitors (EMPs) inside the yolk sac (YS) bypass monocytic intermediates and give rise to microglia via the transcription element *c-Myb*. Ultimately, *c-Myb*^+^ EMPs migrate into the fetal liver (FL), giving rise to fetal liver monocytes that migrate to fetal tissues to generate various types of mature macrophages^10^. Therefore, hematopoietic stem cell-independent embryonic precursors that transiently present in the YS and FL represent the origin of long-lasting, self-renewing resident macrophage populations with organ-specific functions^1,6^ (Fig. 1).

Resident macrophages vary according to their origin and the developmental stage of the organism in each tissue type. For instance, most microglia in the brain originate from YS-derived macrophages, with progenitors from the FL and BM showing a negligible contribution in all stages of life; however, they contribute to other tissue macrophages^2^. For example, resident macrophages in the gut are derived from embryonic precursors (YS macrophages and FL monocytes) at some point during early embryonic development, with subsequent replacement by BM-derived monocytes. FL-derived monocytes give rise to most resident macrophages in the intestine at birth; however, throughout adulthood, most resident macrophages originate from the BM^2^. The percentages of YS-, FL-, and BM-derived macrophages have been revealed using parabiosis and genetic fate-mapping approaches^11^. Using BM-derived monocyte fate mapping, Liu et al., identified the membrane-spanning 4-domains, subfamily A, member 3 (Ms4a3) as a specific gene for faithfully tracking granulocyte-monocyte progenitors (GMPs) and their progeny, which include monocytes^12^. They generated tdTomato reporter and *Ms4a3*^*tdT*^ fate-mapper models to specifically dissect monocyte differentiation pathways, followed by monocytes and their progenies, and to quantify their contribution to the tissue-resident macrophage pool during homeostasis and inflammation^12^.

The dynamics of macrophage populations in the developing cochlea have been mostly characterized in mice and are summarized in Fig. 1^8,9^. Colony-stimulating factor 1 (Csf1) signaling controls the seeding of a larger macrophage population within the cochlea throughout development^8,13^. A second population derived from the FL in a Csf1 receptor (Csf1r) -independent manner is observed in the modiolus and intraluminal surface of the perilymphatic area inside the embryonic cochlea^8^. Under normal conditions, perivascular macrophages (PVMs) lie close to the blood vessels of the stria vascularis in the adult cochlea^14^ (Supplementary Fig. S1). Further, macrophages are present in other regions such as the cochlear modiolus, spiral ganglion neurons, and spiral ligament^8,9,13–17^, with their population being continuously replaced by the supply from bone marrow hematopoiesis in the mature adult cochlea^9,17^(Supplementary Fig. S1).

Around postnatal day 14 (P14) in the mouse cochlea, peripheral auditory perception begins to work once the cochlear sensory epithelium is differentiated and the blood-labyrinthine barrier is formed^18^. During the period of the onset of auditory function, drastic changes should occur in the cochlea homeostasis, with the crucial involvement of tissue-resident macrophages and migrating monocytes. However, the origin of resident macrophages during the period between the formation of the blood-labyrinthine barrier and the onset of auditory function in the postnatal and early adult cochlea remains unclear. Thus, this study aimed to investigate the transformation of tissue-resident macrophages substituted by BM-derived monocytes during the phases of postnatal immaturity and early adulthood in the inner ear.

## Results

### Migration of circulating monocytes as resident macrophages into the postnatal and matured cochlea

**Figure 2:**
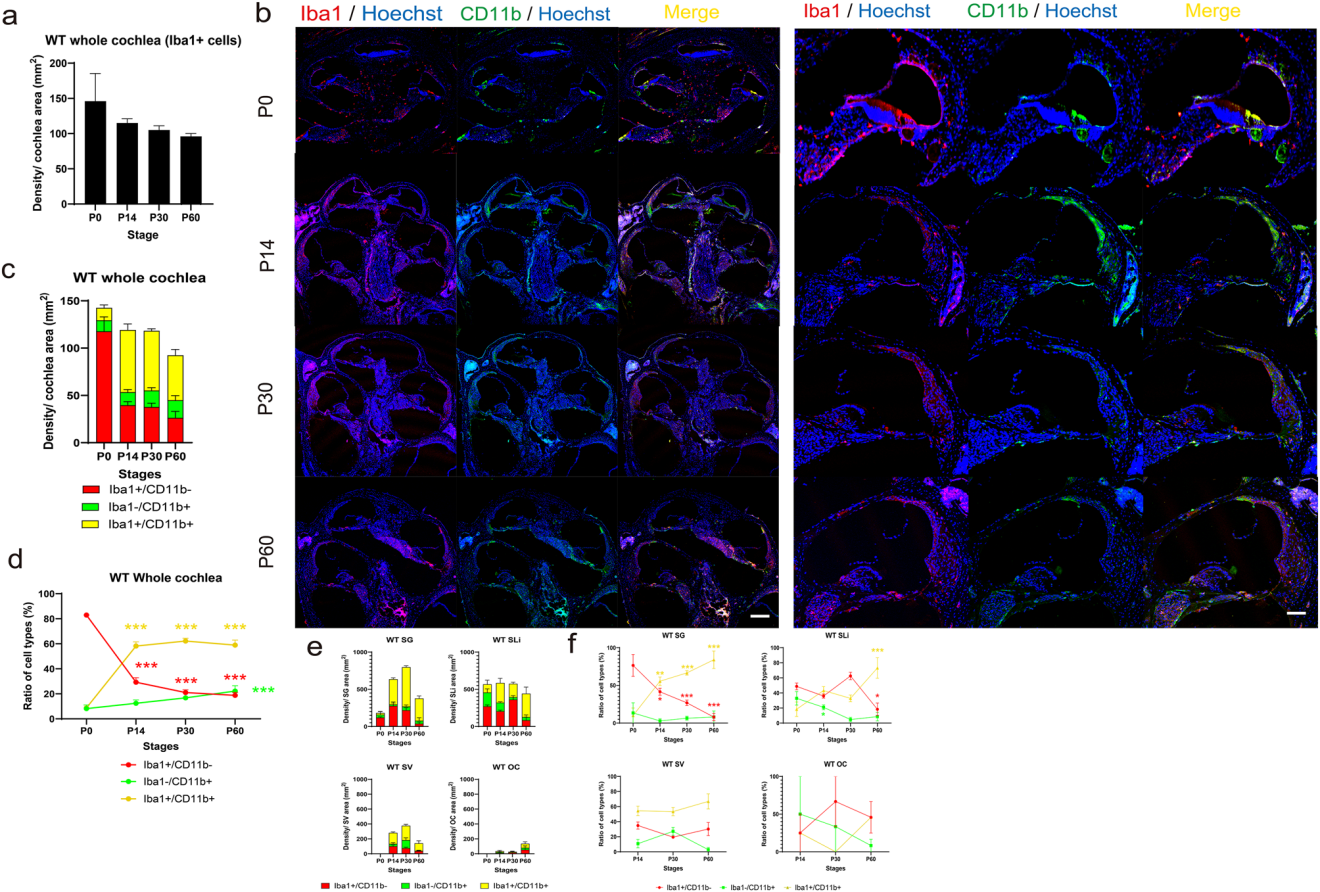
Immunostaining for Iba1 and CD11b and the time course of density in wildtype mice. (**a**) Density of Iba1 + cells at each stage. (**b**) Immunostaining of wildtype mice cochlea of Iba1 and CD11b antibodies at each stage. (Left) Whole cochlea section. (Right) Cochlea sections in the middle turn. Immunostaining for Iba1 and CD11b showed a rapid decrease from P0 to P14 followed by a gradual decrease from P14 to P30 in the number of Iba1^+^/CD11b^−^ cells. In contrast, there was an increase in the number of Iba1^−^/CD11b^+^ and Iba1^+^/CD11b^+^ cells with age. Red, Iba1; green, CD11b; blue, Hoechst nuclei; yellow, merged images. (**c**) Density at each stage. (**d**) Percentages of Iba1^+^/CD11b^−^, Iba1^−^/CD11b^+^, and Iba1^+^/CD11b^+^ cells at each stage. (**e**) Density in each organ at each stage. (**f**) Percentages of Iba1^+^/CD11b^−^, Iba1^−^/CD11b^+^, and Iba1^+^/CD11b^+^ cells in each organ at each stage. Red, Iba1; green, CD11b; yellow, double-positive cells. Asterisks represent statistical significance when *p*-values are < .05 (two-way ANOVA). Scale bars: 100 µm. Iba1, ionized calcium-binding adapter molecule 1. SG, spiral ganglion; SLi, spiral ligament, SV, stria vascularis; OC, organ of Corti.

A previous study demonstrated a clear contrast between subtypes of Iba1-positive (Iba1^+^/CD11b^−^) and CD11b-positive and double-positive cells (Iba1^−^/CD11b^+^ and Iba1^+^/CD11b^+^, respectively)^8^, indicating distinct roles of each subpopulation. At least at P0, Iba1^+^/CD11b^−^ cells indicated YS-derived tissue-resident macrophages, while Iba1^−^/CD11b^+^ cells indicated FL- and BM-derived macrophages or monocytes migrating from systemic circulation^8^. However, the specific characteristics of these cell populations at P14–60 remain unclear. To reveal alterations with age in these three subpopulations, we performed immunostaining for Iba1 and CD11b in the wildtype cochleae at P0 (immature inner ear), P14 (onset of auditory function), P30 (young mice), and P60 (adult mice). CD11b is used as a marker for macrophages originating from FL-derived precursors, BM-derived circulating monocytes, or dendritic cells^10^. Immunostaining for Iba1 and CD11b showed a rapid decrease from P0 to P14; subsequently, there was a gradual decrease from P14 to P30 in the number of Iba1^+^/CD11b^−^ cells and an increase in the number of Iba1^−^/CD11b^+^ and Iba1^+^/CD11b^+^ cells with age (Fig. 2a–c). The density ratio of Iba1^+^/CD11b^−^ cells in the whole cochlea showed 0.35-, 0.25-, and 0.22-fold decreases at P14, P30, and P60, respectively, compared with that at P0 (two-way ANOVA, all *p* < 0.001, Fig. 2c,d, Table 1). The density ratio of Iba1^−^/CD11b^+^ cells in the whole cochlea showed 1.51-, 2.03-, and 2.68-fold increases at P14, P30, and P60, respectively, compared with that at P0 (P0 vs. P60; *p* < 0.001, Fig. 2c,d, Table 1). The density ratio of Iba1^+^/CD11b^+^ cells in the whole cochlea showed 6.61-, 7.06-, and 6.69-fold increases at P14, P30, and P60, respectively, compared with that on P0 (all *p* < 0.001, Fig. 2c,d, Table 1). Taken together, these findings revealed ongoing dynamic changes in the subpopulation of macrophages/monocytes even in the postnatal and young-adult cochlea. In addition, we investigated the density ratio of Iba1^+^/CD11b^−^, Iba1^−^/CD11b^+^, and Iba1^+^/CD11b^+^ cells in each inner ear region. There was a gradual decrease in the number of Iba1^+^/CD11b^-^ cells with age in all four regions. Contrastingly, the ratio of the three types of cells differed across the spiral ganglion (SG), spiral ligament (SLi), stria vascularis (SV), and area beneath the organ of Corti (OC) (Fig. 2e,f, Supplementary Fig. S2). In the SG, the density ratio of Iba1^+^/CD11b^−^; Iba1^−^/CD11b^+^; and Iba1^+^/CD11b^+^ cells at P14, P30, and P60 showed 0.54-, 0.35-, and 0.10-; 0.21-, 0.48-, and 0.59-; and 5.55-, 6.66- and 8.4-fold changes; respectively, compared with that at P0 (Iba1^+^/CD11b^-^: P14, *p* = 0.02; P30 and P60, *p* < 0.001; Iba1^+^/CD11b^+^: P14, *p* = 0.002; P30 and P60, *p* < 0.001; Fig. 2e,f, Supplementary Fig. S2). In the SLi, the density ratio of Iba1^+^/CD11b^−^; Iba1^−^/CD11b^+^; and Iba1^+^/CD11b^+^ cells at P14, P30, and P60 showed 0.74-, 1.28-, and 0.37-; 0.64-, 0.14-, and 0.26-; and 2.36-, 1.78- and 3.99-fold changes; respectively, compared with that at P0 (Iba1^+^/CD11b^−^: P60, *p* = 0.02; Iba1^−^/CD11b^+^: P30, *p* = 0.03; Iba1^+^/CD11b^+^: P60, *p* < 0.001; Fig. 2e,f, Supplementary Fig. S2). In the SV, the density ratio of Iba1^+^/CD11b^−^; Iba1^−^/CD11b^+^; and Iba1^+^/CD11b^+^ cells at P30 and P60 showed 0.55- and 0.86-; 2.51- and 0.26-; and 0.98- and 1.23-fold changes; respectively, compared with that at P14 (Fig. 2e,f, Supplementary Fig. S2). In the OC, the density ratio of Iba1^+^/CD11b^−^; Iba1^-^/CD11b^+^; and Iba1^+^/CD11b^+^ cells at P30 and P60 showed 2.66- and 0.68-; 0.67- and 0.25-; 0- and 0- fold changes, respectively, compared with that at P14 (Fig. 2e,f, Supplementary Fig. S2). These data suggested that resident macrophages are repopulated and replaced in an organ- or region-specific manner in the postnatal and young adult mouse cochlea. Circulating monocyte-derived cells of FL or BM origin are supplied after birth, and their proportion increases to approximately 80% of the total resident macrophages. Regarding morphology, most Iba1 + /CD11b + cells were round-shaped at P0, indicating immaturity or naivety. However, at P14, P30, and P60, Iba1^+^/CD11b^+^ cells in the SV and the area beneath OC were generally round-shaped, while those in the SG and SLi exhibited a spindle shape with elongated processes suggesting mature and surveilling cells^19^.

**Table 1:** Percentages of Iba1^+^/CD11b^−^, Iba1^−^/CD11b^+^, and Iba1^+^/CD11b^+^ cells or Iba1^+^/Ms4a3^−^, Iba1^−^/Ms4a3^+^, and Iba1^+^/Ms4a3^+^ cells in whole cochlea at each stage.

		P0	P14	P30	P60
WT	Iba1 + /CD11b−	82.9	29.2	21.0	18.8
	Iba1−/CD11b +	8.2	12.4	16.7	22.1
	Iba1 + /CD11b +	8.8	58.2	62.2	58.9
Ms4a3 ^tdT^	Iba1 + /Ms4a3−	94.2	75.6	58.2	40.2
	Iba1−/Ms4a3 +	2.6	4.3	8.5	12.5
	Iba1 + /Ms4a3 +	3.1	20.0	33.2	47.1
Csf1r-deficient	Iba1 + /CD11b−	27.9	6.62	1.93	2.42
	Iba1−/CD11b +	38.9	51.2	53.7	41.2
	Iba1 + /CD11b +	33.1	42.1	44.2	56.3

*WT* wild type.

### Contribution of bone marrow hematopoiesis to the macrophage population in postnatal development of the cochlea

**Figure 3:**
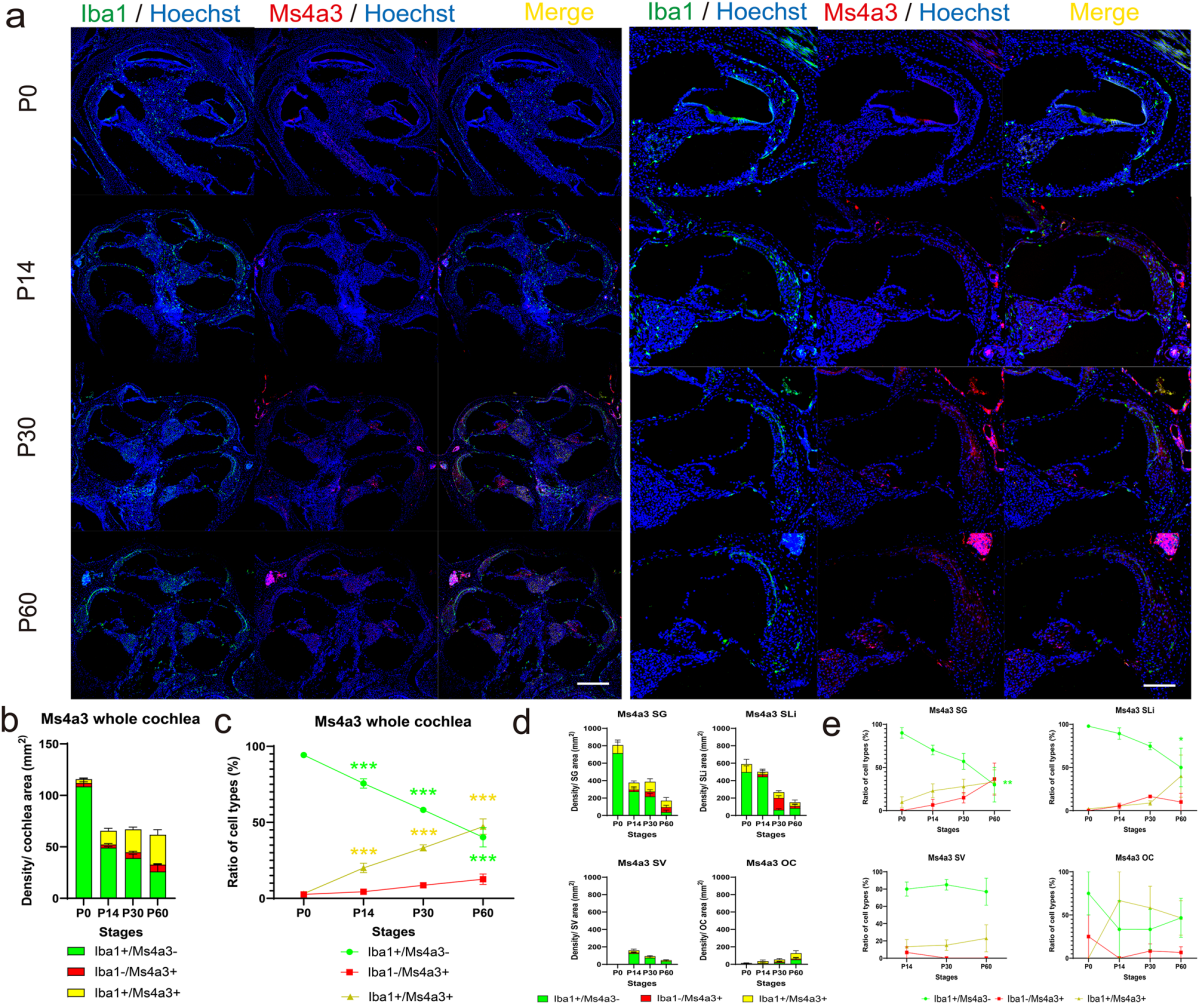
Immunostaining for Iba1 and time course of density in *Ms4a3Cre-Rosa tdTomato*-transgenic mice. (**a**) Immunostaining of the Iba1 antibody and tdTomato reporter in *Ms4a3Cre-Rosa tdTomato* (*Ms4a3*^*tdT*^) transgenic mice cochlea at each stage. (Left) Whole cochlea section. (Right) Cochlea sections in the middle turn. Fate mapping of monocytes labeled with Ms4a3 clearly revealed that the proportion of bone marrow-derived cochlear macrophages increased in the adult stage, whereas the proportion of yolk sac- and fetal liver-derived tissue-resident macrophages decreased steadily with age. Red, tdTomato; green, Iba1; blue, Hoechst nuclei; yellow, merged images. (**b**) Density at each stage. (**c**) Percentages of Iba1^+^/Ms4a3^−^, Iba1^−^/Ms4a3^+^, and Iba1^+^/Ms4a3^+^ cells at each stage. (**d**) Density in each organ at each stage. (**e**) Percentages of Iba1^+^/Ms4a3^−^, Iba1^−^/Ms4a3^+^, and Iba1^+^/Ms4a3^+^ cells in each organ at each stage. Red, tdTomato; green, Iba1; yellow, double-positive cells. Asterisks represent statistical significance when *p*-values are < .05 (two-way ANOVA). Scale bar: 100 µm. Iba1, ionized calcium-binding adapter molecule 1. SG, spiral ganglion; SLi, spiral ligament, SV, stria vascularis; OC, the organ of Corti.

To elucidate the contribution of BM hematopoiesis to the supply of macrophage/monocyte lineage in the postnatal and adult cochlea, we applied lineage tracing of BM-derived cells and utilized *Ms4a3*^*tdT*^ transgenic mice, which can be used to visualize GMP-derived cells and their progenies that include monocytes. In the cochlear sections in *Ms4a3*^*tdT*^ transgenic mice, the density of tdTomato-positive (Ms4a3^+^) cells showed a gradual increase with age, which contrasted with the decreased density of Iba1^+^/Ms4a3^−^ cells (Fig. 3a,b). The density ratio of Iba1^+^/Ms4a3^+^ cells in the whole cochlea showed a substantial increase compared with that at P0 (P14; 6.45-fold, *p* = 0.002, P30; 10.7-fold, *p* < 0.001, P60; 15.2-fold, *p* < 0.001, Fig. 3b,c, Table 1). The density ratio of Iba1^−^/Ms4a3^+^ cells increased at each age compared with that at P0; however, the differences were not statistically significant (Fig. 3b,c, Table 1). The density of Ms4a3^+^ cells showed a gradual increase with age, showing the contrast with a decreased density of Iba1^+^/Ms4a3^−^ cells in the SG and SLi compared with those at P0 (SG: P60; 0.33-fold, *p* = 0.001, SLi: P60; 0.61-fold, *p* = 0.01). However, age-related alterations in the three types of cells were quite different in the SV and the area beneath OC (Fig. 3d,f, Supplementary Fig. S2). An increase in the density of Iba1^−^/Ms4a3^+^ cells suggested that BM-derived monocytes actively migrated in the normal postnatal and young adult cochlea. Additionally, the replacement of Iba1^+^ cells by Iba1^+^/Ms4a3^+^ cells indicated that resident macrophages in the neonatal and young adult cochlea are repopulated by a continuous supply of BM-derived macrophages. The percentage of Ms4a3-positive cells reaches more than half at P60 in the SLi, SG, and the area beneath OC; however, YS- and FL-derived embryonic resident macrophages were still maintained throughout early adulthood. On the other hand, unlike in other sites such as the SLi or SG, the proportion of BM-derived macrophages in the SV remained at a low level even at P60. This indicated that YM- or FL-derived embryonic macrophages are maintained after P14 when the blood-labyrinthine barrier is formed. Considering the ratio of Iba1/CD11b phenotypes in wildtype ICR mice shown in Fig. 2, YM- or FL-derived embryonic macrophages are expected to have a high contribution to the macrophage population in the SV of early adult stages where the blood-labyrinthine barrier strictly restricts the entry and exit of substances and cells. Taken together, these findings indicate that BM hematopoiesis contributes to the macrophage population in postnatal and early adult cochleae.

### Hematopoiesis in the fetal liver and bone marrow contributes to the repopulation of cochlear macrophages with age despite the depletion of YS-derived macrophages in the cochlea

**Figure 4:**
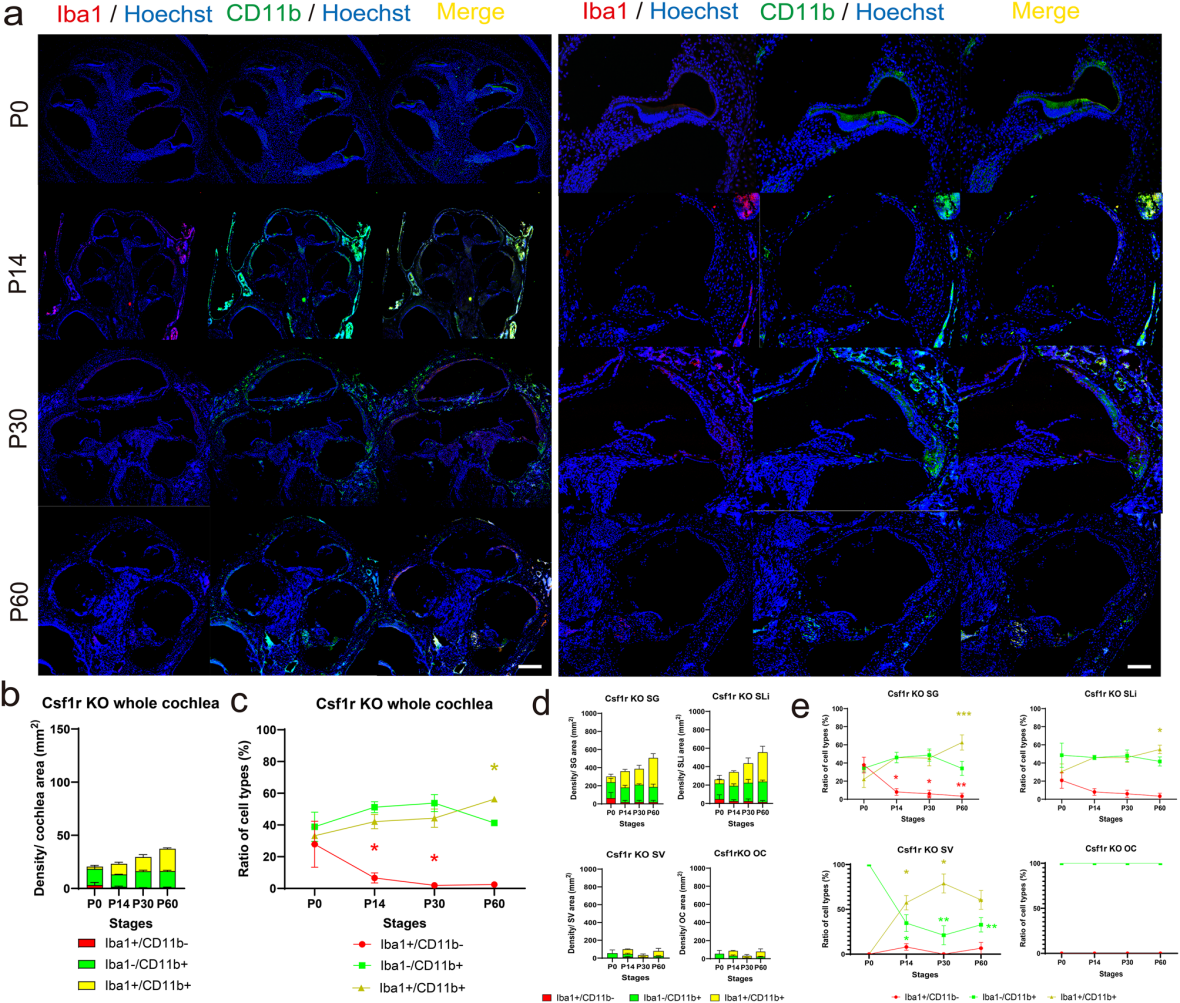
Immunostaining for Iba1 and CD11b and the time course of density in *Csf1r*-deficient mice. (**a**) Immunostaining with Iba1 and CD11b antibodies in *Csf1*-deficient mice cochlea at each stage. (Left) Whole cochlea section. (Right) Cochlea sections in the middle turn. Hematopoiesis in the fetal liver and bone marrow contributes to the repopulation of cochlear macrophages with age despite depletion of yolk sac-derived macrophage in the cochlea of *Csf1*-deficient mice. Red, Iba1; green, CD11b; blue, Hoechst nuclei; yellow, merged images. (**b**) Density at each stage. (**c**) Percentages of Iba1^+^/CD11b^−^, Iba1^−^/CD11b^+^, and Iba1^+^/CD11b^+^ cells at each stage. (**d**) Density in each organ at each stage. (**e**) Percentages of Iba1^+^/CD11b^−^, Iba1^−^/CD11b^+^, and Iba1^+^/CD11b^+^ cells in each organ at each stage. Red, Iba1; green, CD11b; yellow, double-positive cells. Asterisks represent statistical significance when *p*-values are < .05 (two-way ANOVA). Scale bars: 100 µm. Csf1r, colony-stimulating factor 1 receptor; Csf*1r*-KO, *Csf1r*-deficient; Iba1, ionized calcium-binding adapter molecule 1; SG, spiral ganglion; SLi, spiral ligament, SV, stria vascularis; OC, the area beneath the organ of Corti.

As previously reported, YS hematopoiesis in embryonic mice largely depends on Csf1 signaling, resulting in a striking decrease in the number of tissue-resident macrophages, including cochlear macrophages in *Csf1r*-deficient mice^8^. To examine the repopulation of tissue-resident macrophages in the postnatal and adult cochleae when YS hematopoiesis is diminished, we performed immunostaining for Iba1 and CD11b in the cochleae of *Csf1r*-deficient mice. There was a dramatic decrease in the total number of macrophage/monocyte lineage cells and the density of Iba1^+^ cells in all development stages (Fig. 4a,b). Contrastingly, the density of both Iba1^−^/CD11b^+^ and Iba1^+^/CD11b^+^ cells increased with age (Fig. 4b,c); however, the density of macrophages in the absence of YS-derived macrophages was not compensated to the level observed in wildtypes at P60 (Fig. 2b). Moreover, there was an increase in the density ratio of Iba1^−^/CD11b^+^ and Iba1^+^/CD11b^+^ cells in the whole cochlea compared with that at P0 (Iba1^+^/CD11b^+^, P60: 1.69-fold, *p* = 0.04; Fig. 4b,c, Table 1). These data suggest that defects in the resident macrophage population in *Csf1r*-deficient mice are covered and rescued, at least partially, by hematopoiesis in the FL and BM, in addition to the supply of macrophages via systemic circulation. We also investigated the density ratio in each region of the SG, SLi, SV, and OC in the inner ear. The density of Iba1^+^/CD11b^+^ cells gradually increased with age in the SG, SLi, and SV compared with that at P0 (SG: P60; 6.26-fold, *p* < 0.001, SLi; P60; 5.49-fold, *p* = 0.04, SV: P14, P30, P60; all *p* < 0.001). However, there was no consistent trend in cell density changes in the area beneath the OC (Fig. 4d,e, Supplementary Fig. S2). Specifically, in the SV, there were no Iba1-positive macrophages in *Csf1r*-deficient mice in P0; contrastingly, there were only a few CD11b-positive cells residing in the SV. Additionally, there were no drastic changes in the Iba1/CD11b subtype ratio after P14 when the blood-labyrinthine barrier formed, which restricted the migration of circulating cells. These data also suggest that macrophages in the SV originate from the YS and FL and that BM-derived cells contribute minimally to the macrophage population in the SV of a young adult cochlea.

## Discussion

The present study investigated the spatial and temporal distribution patterns of resident macrophages in the cochlea from P0 through P60 in the postnatal and early adult stages. Our previous findings on the in situ proliferative capacity of Iba1-positive cochlear macrophages suggest that the high density of macrophages in the neonatal cochlea may be attributable to in situ proliferation rather than recruitment of macrophages from the systemic circulation. However, during the adult stages, there was an increased rate of monocytes recruited from the systemic circulation; moreover, Iba1^+^/CD11b^−^ or Iba1^+^/Ms4a3^−^ cochlear macrophages gradually decreased with age. These findings suggested the aging of the immune system as previously shown^20^, Additionally, we studied the immunohistochemistry for both Iba1 and CD11b to assess the distribution of resident macrophages and precursors derived from systemic circulation in the cochlea. Iba1^−^/CD11b^+^ cells are considered precursors of tissue-resident macrophages derived from the FL or BM in the adult cochlea^8,21^. There were distinct differences in the distribution of Iba1^+^/CD11b^−^ and Iba1^−^/CD11b^+^ cells, indicating that Iba1^+^/CD11b^−^ cells are mainly distributed in the SG and SLi. In comparison, Iba1^−^/CD11b^+^ cells are predominantly seeded in the mesenchyme of the cochlear modiolus along the auditory nerve. These findings are consistent with previous findings that cochlear resident macrophages in adult mice were slowly replaced by circulating monocyte precursors supplied through hematopoiesis in the BM^9^. Cochlear macrophages persist from the early postnatal stages and renew or preserve their population via infiltration of circulating monocytes^11,15,22^. Additionally, our findings indicate a decrease in most tissue-resident macrophages established in the embryonic stage, and BM-derived macrophages were replaced in the postnatal and early adult cochlea. Interestingly, most resident macrophages in the SV were considered to have originated from YS- or FL-derived macrophages, with BM-derived macrophages showing less contribution in this region during the early adult stage unlike in other cochlear regions such as the SG or SLi. These findings indicate that the existence of the blood-labyrinthine barrier restricts the migration of BM-derived circulating monocytes into the SV P14 onwards. The resident macrophages in the SV, PVMs, are found adjacent to the blood vessels of the intermediate layer of the SV^16^. PVMs slowly proliferate from 3 to 14 days in normal mice; however, in irradiated mice, most PVMs are turned over via BM-cell migration within a 10-month time frame^17^. Functionally, PVMs play an active role in maintaining the dynamic permeability of the blood-inner ear barrier^22^, and contribute to a small extent to the restoration of endocochlear potential^23,24^.

The proportion of macrophages originating from the YS, FL, and BM can change depending on the life stage^2^. Using flow cytometry, quantitative analyses showed that alveolar fetal monocytes differentiate into mature alveolar macrophages with decreasing CD11b and increasing F4/80 expression^2^. Notably, the proportions of macrophages in the cochlea were not consistent with those in the brain, which was previously considered to have a similar construct as the inner ear or eye. The cochlea has a blood labyrinth barrier; therefore, it is considered to be in a region limited from the systemic immune system^25^. However, our findings suggest that the cochlea is a semi-open region similar to the heart, pancreas, gut, and dermis concerning systemic immunity or maintenance of resident macrophages. These findings indicate that the immune system functions in the cochlea and that immunotherapy for inner-ear diseases could be developed through macrophages that can migrate into the inner ear. Furthermore, the cochlea is a unique tissue compared with those found in the heart, pancreas, gut, or dermis, since resident macrophages in the cochlea have a trend of moderate turnover and replacement by BM hematopoiesis. The difference among organs or tissues may be attributable to age-related changes; however, further studies are warranted.

The inner ear plays a crucial role in both auditory sensation and the perception of acceleration/rotation. Unfortunately, replenishing the population of sensory cells within the inner ear after degeneration resulting from acute or chronic injuries is a challenging task. This difficulty is attributed to the fact that, in humans, the hair cells in the inner ear undergo terminal differentiation and lose their capacity for self-renewal in cases where significant damage occurs post-birth^26^. SG neurons, which mediate synaptic connections between the hair cells and neurons of the cochlear nucleus in the brainstem, also undergo damage and degeneration; additionally, damage or atrophy of the SV and SLi disrupts cochlear function. Repeated exposure of the auditory system to insults, including hyperacoustic stimulus or ototoxic drugs, harms these structures, resulting in functional impairments that lead to progressive hearing loss^26^. Apart from the resident macrophages at the damaged site, circulating monocytes are continuously recruited to meet the demands of the inflammatory response as well as the expression of chemokines, cytokines, and cell adhesion molecules. Identifying and establishing a set of markers expressed by cochlear macrophages through multiplex and comparative transcriptomic studies may improve our ability to identify sub-populations and roles as well as to compare the profiles of resident macrophages in different organs. To elucidate the roles of these cells, numerous processes that permit source- and area (cochlear substructure)-specific macrophage analyses are required. Furthermore, these techniques can be used to identify remote cells or tissue indicators in situ. For example, infiltrating monocytes exhibit functional variations in the brain and contribute to disease pathology via multiple mechanisms following ischemic stroke and multiple sclerosis^27^. An alternative monocyte-based therapeutic approach for inner ear pathology might be to facilitate the phagocytosis of loaded delivery vehicles by monocytes, which then passively target the disease site due to the mounting immune response.

This study has several limitations. First, although we determined the proportions of the three macrophage types, we did not elucidate the role of each macrophage type. Second, we selected time points of P0, P14, P30, and P60; however, the later aging state was not examined due to a lack of adequate samples. In addition, a few images were not consistent with the quantitative data. While we tried to match the images with quantitative data, some of the images were not typical. Much sampling may have been necessary. Future studies are warranted to investigate older-stage macrophages using *Ms4a3* transgenic mice. Finally, there were some differences in the total numbers and densities of macrophages among wildtype ICR mice, *Ms4a3* transgenic mice, and *Csf1r-*deficient mice. This could be attributed to the differences in mice strains.

In conclusion, fate mapping of monocytes in *Ms4a3* transgenic mice clearly revealed the proportion of cochlear macrophages in the adult stage. BM-derived macrophages increased during the adult stage, whereas YS- and FL-derived tissue-resident macrophages decreased steadily with age. The origin of the macrophage population in the SV showed a unique profile compared with other cochlear regions, including the SG and Sli. The mechanisms underlying this heterogeneity could be attributed to differences in environmental niches within the tissue or at the sub-tissue level. Future studies are warranted to investigate the role of cochlear macrophages in homeostasis, inflammation, and other diseases, including infection, cancer, and metabolic diseases.

## Methods

### Ethics statements

All experimental protocols were and approved by the Animal Research Committee of Kitano Hospital (approval number: A2010001) and Osaka Metropolitan University (approval number: 22036) and conducted in accordance with the National Institutes of Health (NIH) Guide for the Care and Use of Laboratory Animals and ARRIVE guidelines (https://arriveguidelines.org). Mice were euthanized by cervical dislocation.

### Animals

Wildtype ICR mice were purchased from Clea Japan, Inc. (Tokyo, Japan) on postnatal days (P) 0, P14, P30, and P60. *Csf1r*-deficient mice were kindly provided by Dr. Issay Kitabayashi, National Cancer Center Japan, Tokyo, Japan. The mice were maintained under conventional conditions at the Institute of Laboratory Animals, Kitano Hospital, and Osaka Metropolitan University. The *Ms4a3*^*tdT*^ transgenic mice were kindly provided by Dr. Florent Ginhoux^12^.

### Preparation of frozen sections

Whole heads of neonatal mice at P0 were decapitated under hypothermia anesthesia, immersed in 4% paraformaldehyde in phosphate-buffered saline (PBS) overnight at 4 °C, and cryoprotected with 30% sucrose overnight. The specimens were prepared as cryostat sections (12 µm thick). Midmodiolar sections were used for histological analyses. On P14, P30, and P60, mice under general anesthesia induced by midazolam and medetomidine butorphanol were intracardially perfused with ice-cold PBS, followed by 4% paraformaldehyde in PBS. The temporal bones of the mice were collected and immersed in the same fixative for 4 h at 4 °C. The samples were decalcified with 10% ethylenediaminetetraacetic acid in PBS and cryoprotected with 30% sucrose. The specimens were prepared as cryostat sections (12 µm thick). Midmodiolar sections were used for histological analyses.

### Immunohistochemistry

The cryostat sections were immersed in a blocking solution containing 10% goat serum for 30 min and incubated overnight with a primary antibody at 4 °C. Macrophages were labeled using ionized calcium-binding adapter molecule 1 (Iba1), which is specific for microglia/macrophages, and CD11b, which is specific for the monocyte/macrophage lineage. The primary antibodies used in this study were rabbit anti-Iba1 (1:1000; Wako Pure Chemicals, Osaka, Japan) and rat anti-CD11b (1:500; M1/70; BD Biosciences, San Jose, CA, USA). The localization of primary antibodies was visualized using secondary antibodies conjugated with Alexa Fluor 488 and 594 (1:500; Molecular Probes; Invitrogen, Carlsbad, CA, USA). Cell nuclei were counterstained with Hoechst 33,342 (Invitrogen). The negative controls lacked primary antibody labeling. Fluorescent images were acquired using a TCS SP8 microscope (Leica Microsystems, Wetzlar, Germany). In wildtype, ICR mice and *Csf1r*-deficient mice, Iba1/CD11b double-staining was performed. In *Ms4a3*^*tdT*^ transgenic adult mice, Iba1 staining was performed to visualize the ratio of BM-derived macrophages among the total number of macrophages in the inner ear.

### Calculation of the density of Iba1- or CD11b-positive macrophages

The methods for counting macrophages have been previously described^8^. Briefly, we counted the number of Iba1-, CD11b-, and Ms4a3- positive cochlear macrophages in four sections, which were randomly selected from the 12 most mid-modiolar sections at each postnatal stage by two double-blinded examiners. All Iba1-, CD11b-, and Ms4a3- positive cochlear macrophages within the OC, SG, SLi or SV from the mid-basal portion of the cochlea were counted. The SG, SL, and SV profiles were then traced under a bright field image to generate the area of SG with Image J software (NIH, Bethesda, MD, USA). The density of each portion was expressed as the number of cells per 10,000 μm^2^. The density rate was calculated as the percentage of macrophages in the entire cochlea, converted to 100%.

### Statistical analysis

Statistical analysis was performed using GraphPad Prism software (Prism 9.5.1 for Windows; GraphPad Software Inc., San Diego, CA, USA). A two-way analysis of variance with Tukey’s multiple comparisons test was used to compare the rate of origins of macrophages. Results are presented as mean values ± standard errors. At least five independent samples were prepared and analyzed in each experiment. A two-tailed *p*-value < 0.05 was considered statistically significant.

### Supplementary Information

Supplementary Figures.

## Data Availability

The datasets used and/or analyzed during the current study available from the Toru Miwa on reasonable request.
