# Supplementary material for: Contribution of circulating monocytes in maintaining homeostasis of resident macrophages in postnatal and young adult mouse cochlea
Source: Sci Rep. 2024 Jan 2;14:62. doi: 10.1038/s41598-023-50634-y (PMC10762055; doi:10.1038/s41598-023-50634-y)
Supplement: Supplementary file 1 — Supplementary Figures. [file 41598_2023_50634_MOESM1_ESM.pdf]

SUPPLEMENTARY INFORMATION

# Contribution of Circulating Monocytes in Maintaining Homeostasis of Resident Macrophages in Postnatal and Young Adult Mouse Cochlea

**Toru Miwa[1,2*], Gowshika Rengasamy[3], Zhaoyuan Liu[4], Florent Ginhoux[3,4,5,6], Takayuki Okano[1,7]**

Supplementary Figure 1

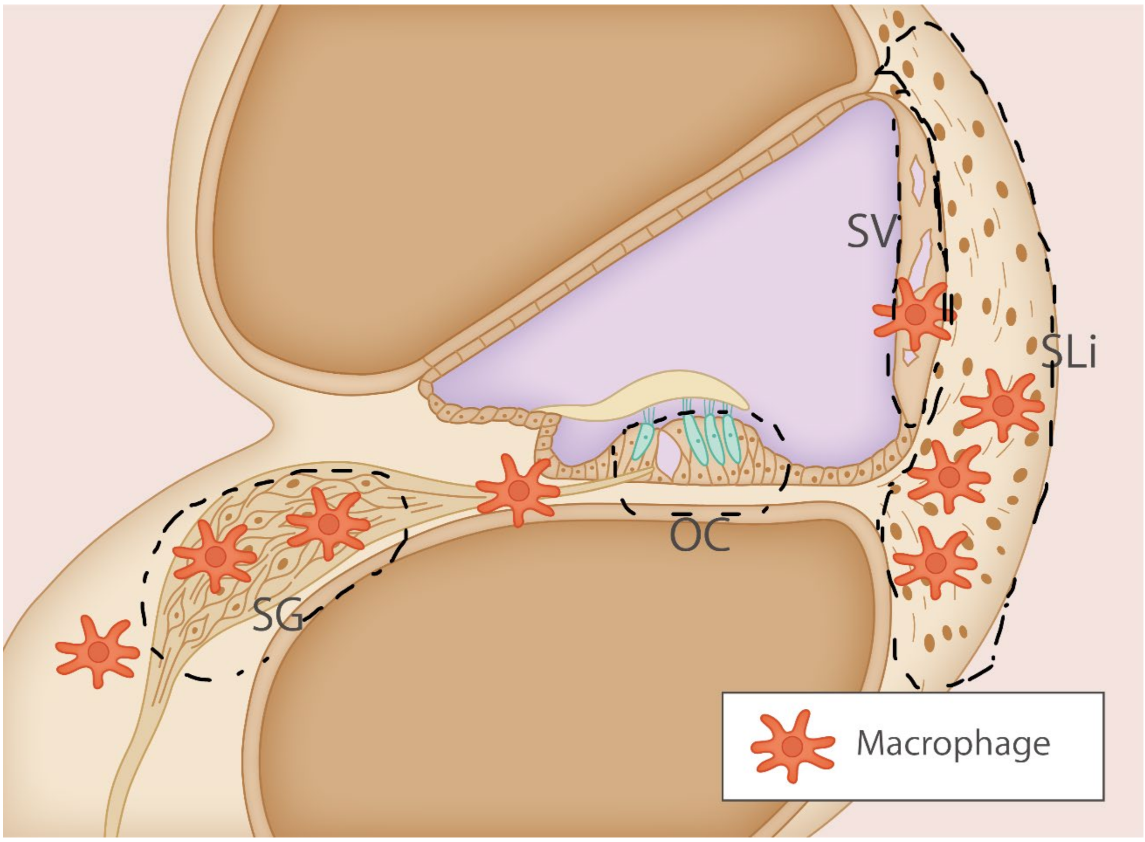


**Supplementary Figure S1.** **Schematic cross-sectional view of the cochlea illustrating the distribution of tissue macrophages.**

Tissue macrophages are distributed in the SG, SLi, SV, OC, and cochlear modiolus. Under steady-state conditions, cochlear macrophages exhibit spindle-shaped cell bodies and dendritic cell processes as do the microglia in the central nervous system. Dashed lines indicated the area of SG, SLi, SV and OC.

SG, spiral ganglion; SLi, spiral ligament, SV, stria vascularis; OC, the area beneath the organ of Corti.

Supplementary Figure S2

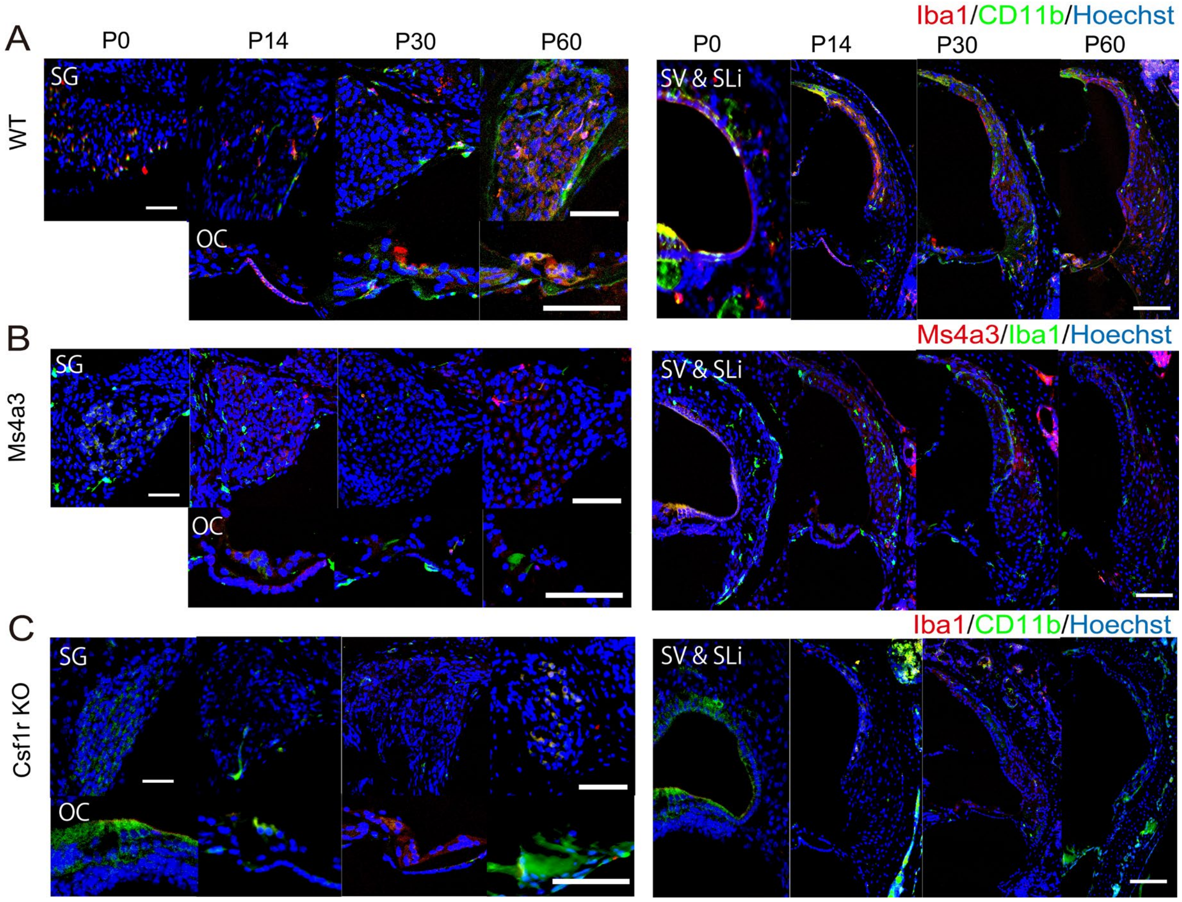


**Supplementary Figure S2. Immunostaining for Iba1 and CD11b and the time course of density in wild-type mice and *Csf1r*-deficient mice and immunostaining for Iba1 and time course of density in *Ms4a3Cre-Rosa tdTomato*-transgenic mice at each organ.**

(a) Immunostaining with Iba1 and CD11b antibodies in wild-type mice cochlea at each stage in each organ in the middle turn. (b) Immunostaining with Iba1 antibody and tdTomato reporter in *Ms4a3Cre-Rosa tdTomato* (*Ms4a3*$^{tdT}$) transgenic mice cochlea at each stage in each organ in the middle turn. Yellow, merged images. (c) Immunostaining with Iba1 and CD11b antibodies in *Csf1r*-deficient mice cochlea at each stage in each organ in the middle turn. Yellow, merged images. Scale bars: 100 µm. Iba1, ionized calcium-binding adapter molecule 1. SG, spiral ganglion; SLi, spiral ligament, SV, stria vascularis; OC, the organ of Corti.
